# Species determination of *Culicoides* biting midges via peptide profiling using matrix-assisted laser desorption ionization mass spectrometry

**DOI:** 10.1186/1756-3305-7-392

**Published:** 2014-08-24

**Authors:** Katrin R Uhlmann, Sebastian Gibb, Stefan Kalkhof, Uriel Arroyo-Abad, Claudia Schulz, Bernd Hoffmann, Francesca Stubbins, Simon Carpenter, Martin Beer, Martin von Bergen, Ralph Feltens

**Affiliations:** Department of Proteomics, Helmholtz-Centre for Environmental Research-UFZ, 04318 Leipzig, Germany; Institute for Medical Informatics, Statistics and Epidemiology (IMISE), University of Leipzig, Härtelstr. 16-18, 04107 Leipzig, Germany; Department of Anesthesiology and Intensive Care, Technical University Dresden, Medical Faculty Carl Gustav Carus, Fetscherstr. 74, 01307 Dresden, Germany; Department of Analytical Chemistry; Reference Materials, BAM-Federal Institute for Materials Research and Testing, Richard-Willstaetter-Str.11, 12489 Berlin, Germany; Department of Analytical Chemistry, Helmholtz-Centre for Environmental Research-UFZ, 04318 Leipzig, Germany; Institute of Diagnostic Virology, Friedrich-Loeffler-Institut, Südufer 10, 17493 Greifswald Insel Riems, Germany; The Pirbright Institute, Pirbright Laboratory, Ash Road, Pirbright, Surrey, GU24 0NF UK; Department of Metabolomics, Helmholtz-Centre for Environmental Research-UFZ, 04318 Leipzig, Germany; Department of Biotechnology, Chemistry and Environmental Engineering, Aalborg University, Sohngårdsholmsvej 49, DK 9000 Aalborg, Denmark

**Keywords:** Culicoides, Species typing, MALDI-TOF-MS, Shotgun mass mapping, MALDIquant

## Abstract

**Background:**

*Culicoides* biting midges are vectors of bluetongue and Schmallenberg viruses that inflict large-scale disease epidemics in ruminant livestock in Europe. Methods based on morphological characteristics and sequencing of genetic markers are most commonly employed to differentiate *Culicoides* to species level. Proteomic methods, however, are also increasingly being used as an alternative method of identification. These techniques have the potential to be rapid and may also offer advantages over DNA-based techniques. The aim of this proof-of-principle study was to develop a simple MALDI-MS based method to differentiate *Culicoides* from different species by peptide patterns with the additional option of identifying discriminating peptides.

**Methods:**

Proteins extracted from 7 *Culicoides* species were digested and resulting peptides purified. Peptide mass fingerprint (PMF) spectra were recorded using matrix-assisted laser desorption/ionization time of flight mass spectrometry (MALDI-TOF-MS) and peak patterns analysed in R using the MALDIquant R package. Additionally, offline liquid chromatography (LC) MALDI-TOF tandem mass spectrometry (MS/MS) was applied to determine the identity of peptide peaks in one exemplary MALDI spectrum obtained using an unfractionated extract.

**Results:**

We showed that the majority of *Culicoides* species yielded reproducible mass spectra with peak patterns that were suitable for classification. The dendrogram obtained by MS showed tentative similarities to a dendrogram generated from cytochrome oxidase I (COX1) sequences. Using offline LC-MALDI-TOF-MS/MS we determined the identity of 28 peptide peaks observed in one MALDI spectrum in a mass range from 1.1 to 3.1 kDa. All identified peptides were identical to other dipteran species and derived from one of five highly abundant proteins due to an absence of available *Culicoides* data.

**Conclusion:**

Shotgun mass mapping by MALDI-TOF-MS has been shown to be compatible with morphological and genetic identification of specimens. Furthermore, the method performs at least as well as an alternative approach based on MS spectra of intact proteins, thus establishing the procedure as a method in its own right, with the additional option of concurrently using the same samples in other MS-based applications for protein identifications. The future availability of genomic information for different *Culicoides* species may enable a more stringent peptide detection based on *Culicoides*-specific sequence information.

**Electronic supplementary material:**

The online version of this article (doi:10.1186/1756-3305-7-392) contains supplementary material, which is available to authorized users.

## Background

*Culicoides* biting midges (Diptera: Ceratopogionidae) have been identified as the primary biological vectors of bluetongue virus (BTV) and Schmallenberg virus (SBV) during recent, unprecedented epizootics of these viruses in northern Europe [1, 2]. These viruses inflict clinical disease in domesticated and wild ruminant host species along with certain species of deer and camelids [3]. The recent emergence of BTV (in 2006) and SBV (in 2011) has demonstrated that there is a potential for further emergence of *Culicoides*-borne pathogens in the future, although the likelihood of this occurring cannot currently be quantified as the route of entry has not been convincingly determined [4].

The reliable identification of *Culicoides* to species level is an important pre-requisite to studying their occurrence and role as vectors, as even closely related species can vary significantly in their ecology. While morphological identification of *Culicoides* can be subjective, time-consuming [5, 6] and may require microscopic dissection and slide-mounting of body parts [7], it remains the technique most commonly employed. Discrimination of cryptic or sibling species and variations within species groups by morphological characteristics, however, is not always achievable [8].

Assays based on the polymerase chain reaction (PCR) have provided an alternative, relatively robust and objective tool for species determination with a high specificity, reproducibility and sensitivity. These assays are based on the sequencing and phylogenetic comparison of mitochondrial or nuclear DNA marker regions, of which the most commonly utilized is the cytochrome oxidase subunit 1 (COX1) gene [9–12]. Additional regions that have been used but in some cases led to conflicting results include the internal transcribed spacer 1 (ITS-1; [13–15]) or 2 (ITS-2; [16, 17]). While a common framework for production and standardization of COX1 marker sequences and voucher specimens has been suggested through the ‘barcode of life’ initiative [18], full compliance with standards set out for submission is rare as a whole and has largely not been achieved for *Culicoides*.

In addition to sequencing, multiplex PCR assays have also been developed in conventional and real-time PCR formats to allow rapid differentiation of *Culicoides* in cryptic species groups [9, 11, 13, 14, 19–23]. These have largely concentrated upon females of the *Avaritia* subgenus, of which *C. obsoletus* and *C. scoticus* in particular are challenging to discriminate by morphology. While these techniques enable significant numbers of *Culicoides* to be processed entirely to species level for certain studies, they are generally limited to those requiring a maximum of several thousand individuals by cost considerations. A potential means of overcoming this limitation may lie in the use of quantitative real-time PCR assays that can be used to define species abundance in homogenized samples [19], although these have yet to be utilized in large-scale studies.

As an alternative molecular technique for the reliable identification of species, detection of peptides and proteins via matrix-assisted laser desorption/ionization time-of-flight mass spectrometry (MALDI-TOF-MS) has emerged during the last decade. In this approach, which is also termed intact protein profiling (IPP), spectra from mixtures of extracted proteins are recorded by MALDI-TOF-MS, utilizing its capability to ionize and measure proteins in the range from 0.5-200 kDa (although in practice the majority of detectable proteins usually lie below 10 kDa). IPP in conjunction with MALDI-TOF-MS has been widely used for the identification of clinically relevant microorganisms [24–31] and for metazoans including plants [32], fish [33] and arthropods [34–39] on the basis of their (predominantly low molecular weight) proteins. In insects, IPP has been successfully applied to the identification of species from the families *Aphididae* [34] and *Culicidae* [40] and the genera *Drosophila* [35, 36], *Anopheles* [37], *Glossina* [41] and *Culicoides* [42]. An IPP-based discrimination of different species has been carried out as well for ticks [38, 39]. The approach is not suitable, however, for sequence analysis and detection of specific proteins by tandem mass spectrometry (MS/MS) and is restricted by its relatively low resolution and limited sensitivity for larger masses.

A complementary method to IPP is peptide mass fingerprinting, also commonly termed shotgun mass mapping (SMM). In this procedure, crude extracts from whole cells or biopsies are subjected to proteolytic hydrolysis by trypsin without any pre-fractionation, and the resulting peptide-containing mixtures may be subjected to MALDI-MS analysis without additional clean-up steps [24]. Spectra recorded by this strategy have been used to detect the presence of cancer in cells [43, 44] or for bacteria species identification [45, 46]. Although analyzing peptides instead of whole proteins requires a somewhat more elaborate sample preparation, this approach offers several advantages over conventional analyses. Firstly, it exploits the high resolution MALDI-MS offers especially in the lower mass range (which can be enhanced even more since for this range the reflector modus of the MS can be used) of 500–4.000 Da relevant for proteolytic peptides, which leads to a significant increase in the number of peaks available for species differentiation. Secondly, the optional ion fragmentation yields sequence-specific spectra, from which species affiliation may be derived if a sufficiently complete genomic dataset for the respective species is available.

Despite the potential use of SMM, so far no study has attempted to use it in the context of species discrimination by using extracts from whole multicellular organisms. This study therefore assesses the feasibility of this approach and additionally evaluates its possible benefits over IPP by differentiating seven *Culicoides* species through MALDI-TOF-MS using peptide mass mapping in a shotgun approach.

## Methods

### Chemicals

All chemicals and solvents were of *pro analysis* quality and purchased from Sigma (Taufkirchen, Germany), Merck (Darmstadt, Germany), Bruker Daltonics (Bremen, Germany) and Bio-Rad (Munich, Germany). High purity water was obtained by an Ultra Clear UV plus system from SG GmbH (Barsbüttel, Germany).

### Culicoides *samples used*, *protein extraction and tryptic digestion*

*Culicoides* were collected as part of a surveillance scheme using light-suction traps conducted in the United Kingdom. Female *Culicoides* of six different species were identified and the laboratory-reared species *C. nubeculosus* was also used during analysis. Samples were stored in 70% ethanol. Prior to sample preparation, every specimen was examined to ensure a lack of physical damage following shipping to the UFZ. For protein extraction, *Culicoides* were transferred individually into reaction tubes and placed in a vacuum centrifuge for 30 to 60 min to remove residual liquid. 20 μL of 7 mol/L (M) urea in 100 mM ammonium bicarbonate [(NH_4_) HCO_3_] was then added to each tube. The *Culicoides* were ground thoroughly with a pestle and the resulting homogenates sonicated with 5 pulses of 0.2 s length and 20% of the maximal amplitude with an UP 50 H lab homogenizer (Hielscher Ultrasonics GmbH, Teltow, Germany). Protein concentrations were then determined using the Quick Start Bradford Protein Assay (Bio-Rad Laboratories GmbH, Munich, Germany).

Reduction and alkylation were carried out by addition of 1 μL of 1 M dithiothreitol (DTT) in 100 mM (NH_4_) HCO_3_ and samples were then incubated for 1 h at 37°C, followed by addition of 20 μL of 200 mM iodoacetamide in 100 mM (NH_4_) HCO_3_ and a further incubation for 1 h in darkness at ambient room temperature. Following addition of 4 μL of 1 M DTT, samples were diluted with 60 μL of 100 mM (NH_4_) HCO_3_. At this point an aliquot of 10 μL of each homogenate was transferred to a reaction tube, mixed with 200 μL RAV1 buffer (NucleoSpin® 96 RNA Kit, Marchery-Nagel, Düren, Germany) and sent to the Friedrich-Loeffler-Institut (FLI) for DNA sequencing.

Specimens for which species affiliation could either not be determined by PCR and sequencing, or where the sequences and entomological analyses provided contradictory results or which apparently contained host blood during sample preparation were deemed unsuitable for analysis and excluded from the study (five individuals in total). 1 μL (specimens belonging to the obsoletus group) or 2.5 μL (specimens belonging to the pulicaris group or *C. nubeculosus*) of 0.1 μg/μL trypsin were added to the residual homogenate. Protein digestions were carried out overnight at 37°C and stopped by addition of 1 μL formic acid (FA; >85%). After removal of insoluble material by centrifugation for 10 s, tryptic peptides were extracted and purified using C_18_-ZipTip pipette tips according to the manufacturer’s instructions, and stepwise elution was performed with 10 μL of 30% and 80% acetonitrile (ACN) containing 0.1% FA. Eluted peptides were vacuum-dried and stored at −20°C prior to analysis. Total preparation time is estimated to be approximately 30 min per sample, with an additional 15 h for overnight incubation.

### DNA extraction, PCR amplification and automated DNA sequencing

Partial COX1 sequences were used to identify *Culicoides* biting midges at species level. For this, 100 μL of the *Culicoides*-RAV1-buffer homogenate were mixed with 100 μL of minimal essential medium with 5% foetal bovine serum. Total DNA from single midges was extracted using High Pure PCR Template Preparation kit (Roche) according to the manufacturer’s instructions and was eluted in 100 μL. Sequences of 507 to 537 bp length of the COX1 gene from individual *Culicoides* were amplified with modified versions of genus-specific (“pan-*Culicoides*”) forward and reverse primers, as described by Dallas *et al*. [10] using the QuantiTect Multiplex PCR NoRox Kit (Qiagen; for primer sequences, see supplementary Additional file 1 Table S1). A total of 5 μL eluted sample was used for the PCR reaction. The thermal profile for amplification was 15 min at 95°C, followed by 42 cycles of 45 s at 95°C, 30 s at 60°C and 35 s at 72°C and a final step of 5 min at 72°C in a Mastercycler epgradient S thermocycler (Eppendorf).

PCR products were visualised using electrophoresis in 1.5% agarose gels by ethidium bromide staining and extracted using the QIAquick Gel Extraction kit (Qiagen) according to the manufacturer’s instructions. The amplicons were sequenced bidirectionally with the previously described primers using the BigDye Terminator v1.1 Cycle Sequencing kit (Applied Biosystems) for dye termination cycle sequencing and were purified with Dye Ex 2.0 Spin kit (Qiagen). Forward and reverse sequences were generated with an ABI 3130 Genetic Analyzer instrument (Applied Biosystems) and aligned using CodonCode Aligner (CodonCode Corporation, version: 4.0.3). Sequences of individual *Culicoides* midges were identified using BLASTn search available via NCBI GenBank, and selected for maximal identity.

### MALDI-TOF-MS

Stored peptide pellets were dissolved in 5 μL (obsoletus group) or 10 μL (pulicaris group or *C. nubeculosus*) of 50% ACN containing 0.1% FA. One microliter aliquots of the peptide solutions were mixed with 1 μL α-Cyano-4-hydroxycinnamic acid (HCCA) matrix in a reaction tube and spotted onto a ground steel MALDI target (Bruker Daltonics). The MALDI matrix solution was prepared by dissolving 5 mg HCCA in 1 mL 60% ACN containing 0.1% trifluoroacetic acid (TFA). Samples were allowed to dry for several minutes before MALDI-TOF-MS measurements were performed. SMM spectra were obtained on a MALDI-TOF/TOF mass spectrometer (Ultraflex III™, using FlexControl software version: 3.0; Bruker Daltonics, Bremen, Germany). The laser was operated at a frequency of 100 Hz. Positive ionization and reflector mode were employed for MALDI-TOF-MS measurements of peptide mixtures with deflection of ions with *m*/*z* less than 450. Spectra from 20,000 laser shots per spot were automatically and cumulatively acquired in the *m*/*z* range from 700 to 4,020 Da. Peptide Calibration Standard II (Bruker Daltonics) was used for external calibration of the mass spectra, resulting in a mass accuracy of generally better than 50 ppm.

An IPP spectrum of one *Culicoides* was obtained using the same MALDI-TOF/TOF mass spectrometer as for the SMM measurements. For comparability, protein extraction was carried out as described in the previous section, with the exception that DTT and IAA were dissolved and added to the reaction tube in 100 mM (NH_4_) HCO_3_ containing 7 M urea to maintain protein denaturing conditions. Proteolysis by trypsin was omitted. Purification and spotting of the protein sample was performed as described above. For MALDI-TOF-MS measurements of the protein mixture, positive ionization and linear mode were employed with deflection of ions with *m*/*z* less than 1,400. Spectra from 10,000 laser shots per spot were automatically and cumulatively acquired in the *m*/*z* range from 1.4 to 16 kDa. Protein Calibration Standard I (Bruker Daltonics) was used for external calibration of the mass spectra.

### LC-ESI-MS/MS

The residual digested homogenate of one *Culicoides* was vacuum-dried and dissolved in 20 μL 0.1% FA. 5 μL of the sample (0.4 μg/μL) were analyzed using reversed-phase nanoscale liquid chromatography tandem mass spectrometry on a NanoAcquity UPLC system (Waters Corporation, Milford, USA) connected to an LTQ-Orbitrap XL ETD (Thermo Fisher Scientific, Waltham, USA) equipped with a TriVersa Nanomate nano-ESI source (Advion, Ithaca, USA) as described recently [47]. Briefly, samples were concentrated on a trapping column (nanoAcquity UPLC column, C18, 180 μm x 20 mm, 5 μm, Waters, Milford, USA) with water containing 0.1% formic acid at a flow rate of 15 μL/min. After 7 min, peptides were eluted onto the separation column (nanoAcquity UPLC column, C18, 75 μm × 150 mm, 1.7 μm, Waters, Milford, USA). Chromatography was performed with 0.1% formic acid in solvents A (100% water) and B (100% ACN), with peptides eluting over 90 min LC-MS runtime with a 2–85% solvent B gradient. The flow rate for separation was 300 nL/min.

For MS analysis, continuous scanning of eluting peptide ions was carried out in a mass range *m*/*z* 300–1,600 with automatic switching to CID-MS/MS mode on the six most intensive ions exceeding an intensity of 3,000. Additionally, for CID-MS/MS measurements, a dynamic precursor exclusion of 3 min was applied.

### LC-MALDI-TOF-MS/MS

For offline nano-HPLC/MALDI MS/MS analyses, the same nano-HPLC and the same gradient were used as for nano-ESI-MS/MS analyses. The eluted samples were fractionated post column (30 s per spot). Fractions were manually spotted into 1 μL of ACN (50%) containing 0.1% FA onto an AnchorChip target (600/384 T F, Bruker Daltonics, Bremen, Germany) and 0.5 μL HCCA (0.7 μg/μL in 85% ACN, 0.1% FA, 1 mM (NH_4_)H_2_PO_4_) were added. MALDI-MS/MS analysis was conducted as described in Kalkhof *et al*. [48]. Briefly, MS spectra for each fraction were acquired in the *m*/*z* range from 700 to 4,020. For each spectrum, 10,000 laser shots were accumulated automatically. Data acquisition and data processing were carried out via FlexControl 3.0 and FlexAnalysis 3.0 software. For all detected peptide signals with a signal-to-noise ratio larger than 10, the spots showing the highest intensity for the respective precursor ion were automatically selected and subjected to MALDI-LIFT TOF/TOF-MS/MS by WarpLC 1.0 (Bruker Daltonics, Bremen, Germany) software. For precursor ion isolation, laser shots were accumulated until either a signal-to-noise ratio (SNR) > 30 or a total of 2,100 shots were obtained. For MS/MS spectra, laser shots were gathered until either 8 fragments achieved an S/N > 20 or 2,100 shots were accumulated.

### LC-ESI-MS/MS data analysis

For protein identification, database searches were carried out against a concatenated target/decoy database, which contains all dipteran species entries of the NCBI database (http://www.ncbi.nlm.nih.gov, 03–2013). Searches were performed using Mascot (version: 2.3.01, Matrixscience, London, UK). For ESI-MS/MS data analysis, Proteome Discoverer (version 1.2, Thermo Scientific) was used as an interface as well as for further analysis such as data filtering based on false discovery rate (FDR) and Mascot score and for protein and peptide grouping. Both the protein and the peptide FDR specification were controlled to be below 0.05 and additionally an ion score cut-off of 20 was applied.

For LC-MALDI-MS/MS runs, Mascot searches were utilized by the Biotools software (Bruker Daltonics, version: 3.0). Based upon the identification results, a final protein list with a controlled FDR below 0.05 was created using the WARP LC software.

For peptide identification, maximum mass deviations of either 10 ppm for ESI-MS, 100 ppm for MALDI-MS, 0.8 u for ESI-MS/MS or 0.5 u for MALDI-MS/MS were set. Furthermore, search parameters were set for detection of peptides with methionine oxidation, N-terminal acetylation (optional modifications) and cysteine carbamidomethylation (static modification) and a maximum of two tryptic missed cleavage sites.

### Data processing

All data processing except the MS/MS data was done in R (version: 3.0.2; [49]). The complete R scripts to reproduce the analysis can be downloaded from http://sgibb.github.io/Culicoides/. The raw spectra data are available from http://dx.doi.org/10.6084/m9.figshare.801878.

### Mass spectrometry data preprocessing

The externally calibrated raw spectra were imported into R using the MALDIquantForeign R package (version: 0.5.1; [50]). Spectra were preprocessed using the MALDIquant R package (version: 1.8; [51]). First, a square root variance-stabilizing transformation combined with a 7-point moving average smoothing was applied and baseline correction was conducted using the TopHat algorithm. Next, to enable intensity comparison between different spectra, Total-Ion-Currents (TIC) were equalized and peak detection was performed. To adjust for *m*/*z*-shifts, especially for different days of recording, the spectra were recalibrated by applying individual quadratic warping functions. The warping functions were obtained by aligning spectra using automatically determined reference peaks (MALDIquant; [50]). To detect monoisotopic peaks, an algorithm based on the artificial average amino acid “averagine” [52] and the isotopic-peak-ratio [53] was employed. The monoisotopic peaks were filtered based on the half-decimal-place-rule (HDPR) [54, 55] to ensure that only peptides were analyzed. For this, the cleaver R package (version: 1.0.0; [56]) was used to *in silico* digest the reference proteome of *Drosophila melanogaster* that was downloaded from the UniProt database [57].

After digestion, the monoisotopic mass of the peptides was calculated by the BRAIN R package (version: 1.8.0; [58]). A robust linear regression provided by the MASS R package (version: 7.3.29; [59]) was used to find the correlation of *m*/*z*-values versus their decimal-places in the relevant mass range of 700 to 4,000 Da. Since the slopes of the regression models of the *Drosophila melanogaster* proteome and the experimental data differed significantly (4.9 × 10^-4^ vs. 5.3 × 10^-4^), the latter was chosen as a basis for the subsequent filtering in order to avoid the inadvertent removal of peptide peaks. The *Drosophila melanogaster* dataset was used to determine a tolerance range containing 98% of all peaks. All peaks outside this defined range (±0.2 u) were removed from the experimental dataset (see Additional file 2 Figure S1). The remaining monoisotopic peaks were binned within a *m*/*z* window of 200 ppm. All peaks occurring in only 1 of 3 technical replicates were removed to reduce false-positive/noise-derived peaks. The technical replicates were averaged for each individual. Finally, a peak matrix and a binary peak matrix were created.

### Unsupervised data analysis

The binary peak matrix was used to calculate pairwise spectra similarities using Dice coefficients [60] provided by the proxy R package (version: 0.4-10; [61]). The Dice similarity coefficients were calculated according to D = 2N_m_/(N_a_ + N_b_), with N_m_ for the number of matching peaks in A and B and N_a_, N_b_ for the total number of peaks in the respective spectra. Subsequently, an unsupervised hierarchical clustering analysis using Ward’s minimum variance method [62] and a bootstrapping analysis (N = 1000) were applied. The binary peak matrix was used for the Principal Component Analysis (PCA) as well. The Principal Component Analysis and the plotting of the main components were done using the vegan R package (version: 2.0-7; [63]).

### Supervised data analysis

With the intention of finding discriminating peaks (*m*/*z*-values) to separate species or taxonomic groups, a linear discriminant analysis was performed. In this case, the shrinkage discriminant analysis (SDA) [64] was chosen because its predictor variables are ranked using correlation-adjusted t-scores (CAT scores) [65], allowing simple and effective ranking of peaks even in the presence of correlation. For the analysis, the peak matrix was entered into the sda R package (version: 1.3.2; [66]). This generates a ranking of discriminating peaks for each species or taxonomic group.

### DNA sequence data analysis

The resulting fasta file of the PCR sequencing results was imported and analyzed using the ape R package (version: 3.0-11; [67]). To create the phylogenetic tree based on the cytochrome c oxidase subunit I (COX1) PCR results, the Kimura distance [68] was calculated and a hierarchical clustering using the unweighted pair group method with arithmetic mean (UPGMA) was performed. This was followed by a bootstrapping analysis (N = 1000).

## Results

To show the applicability of Shotgun Mass Mapping (SMM) for the differentiation of *Culicoides* species, 192 SMM spectra were recorded via MALDI-TOF-MS, using peptide extracts prepared from 64 individual *Culicoides* specimens from 7 different species (see Table 1). Using monoisotopic peak detection, around 400 peaks per spectrum were found on average in the *m*/*z* range between 700 and 4,020 Da. Upon visual inspection, *Culicoides* from the same species generally resulted in similar spectra, but showed distinct patterns when compared to spectra from other species. 7 exemplary spectra from the 7 different *Culicoides* species are shown in Additional file 3 Figure S2. Technical replicates yielded close to identical spectra (data not shown).

**Table 1:** **Abbreviations used for** ***Culicoides*** **species**

***Culicoides***species (No. of specimens used)	Abbreviation
*C. dewulfi* (14)	C_Dew
*C. pulicaris* (10)	C_Pul
*C. newsteadi* (10)	C_New
*C. punctatus* (9)	C_Pun
*C. obsoletus* (8)	C_Obs
*C. nubeculosus* (10)	C_Nub
*C. scoticus* (2)	C_Sco

**Figure 1:**
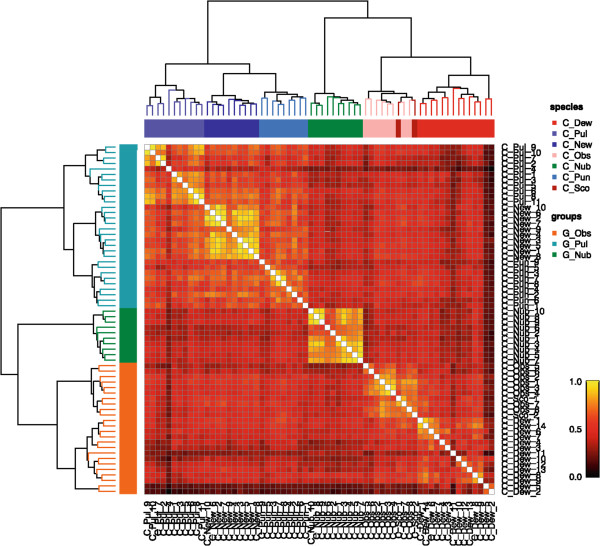
**Results from cluster analysis.** 64 spectra from biting midges of 7 different species were clustered using Dice coefficients. A colour (heatmap) representation of the pairwise similarity (Dice) coefficients is shown. Above this, clustering of individual spectra, color-coded according to species, are shown in a dendrogram. On the left side, the same dendrogram is shown but color-coded according to species group.

**Figure 2:**
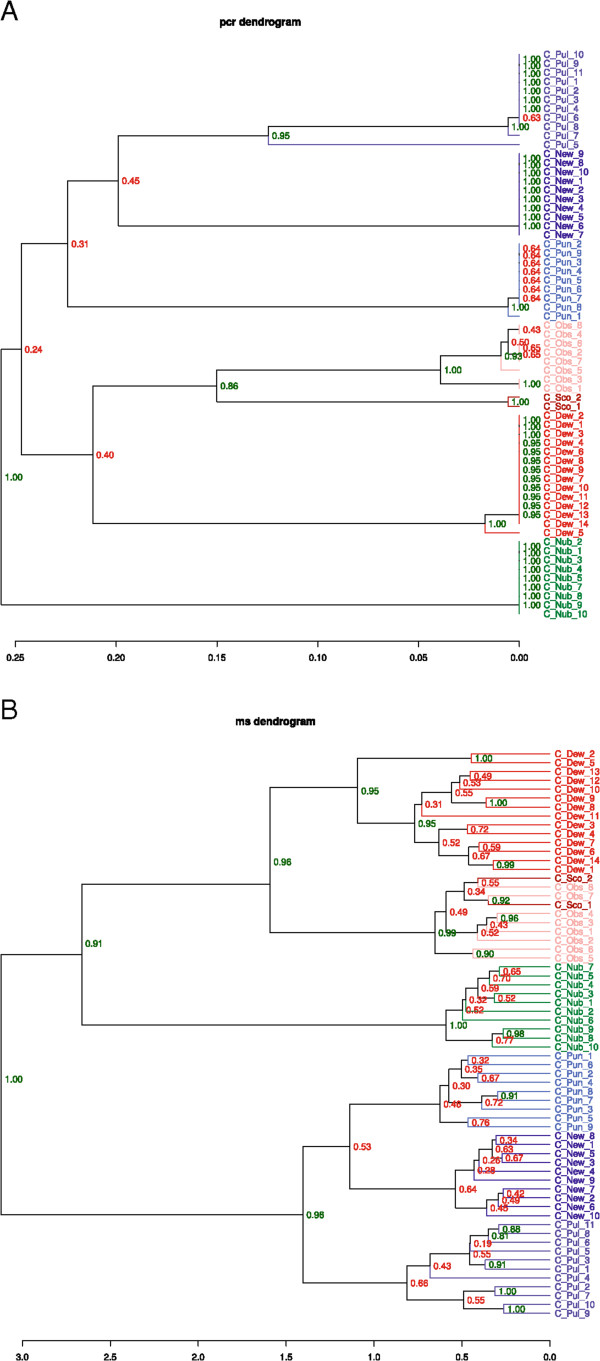
**Comparison of PCR dendrogram (A) and MS dendrogram (B). A**: The PCR dendrogram is based on the COX1 DNA sequencing data, suggesting a possible phylogeny for the different species. **B**: The MS dendrogram is based on the MALDI-TOF MS data. Percentages of bootstrapping replicates supporting the location of individual nodes are indicated.

Data from 2–14 female specimens of each species that had been morphologically identified and corroborated by PCR-analyses (n = 64), were used for a hierarchical cluster analysis, yielding a dendrogram and a similarity matrix (Figure 1). Although *C. scoticus* and *C. obsoletus* could clearly be separated from all other species, distinction between spectra from these two species was not achieved using MALDI-TOF-MS. For comparison, possible phylogeny inferred from genomic (partial COX1 gene sequence) and proteomic SMM data is represented by the dendrograms shown in Figures 2A and 2B, respectively.

**Figure 3:**
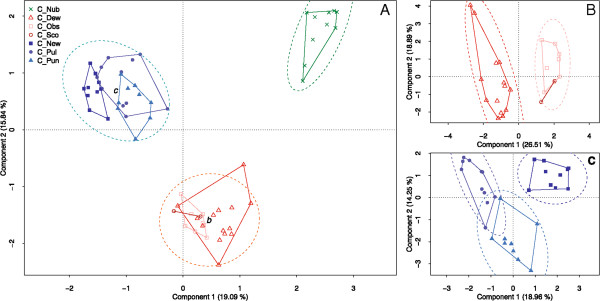
**Scatterplot from unbiased PCAs using 64 spectra from midges of 7 different** ***Culicoides*** **species.** Species-specific colour coding corresponds to that shown in Figure 1. Spectra belonging to one species are outlined by convex shape. The dashed lines indicate 95% concentration ellipses. **A**: PCA containing all spectra; **B** spectra from the obsoletus group only; **C**: spectra from the pulicaris group only.

To evaluate the peak matrix with a different, independent method, a principal component analysis (PCA) of all 64 spectra was performed. Species groups (Figure 3A) as well as individual species within these groups (Figure 3B and C) were distinguishable, with the exception of *C. obsoletus* and *C. scoticus*, which could not be separated, and *C. punctatus* and *C. pulicaris*, which show some overlap in their respective 95% concentration ellipses.

**Figure 4:**
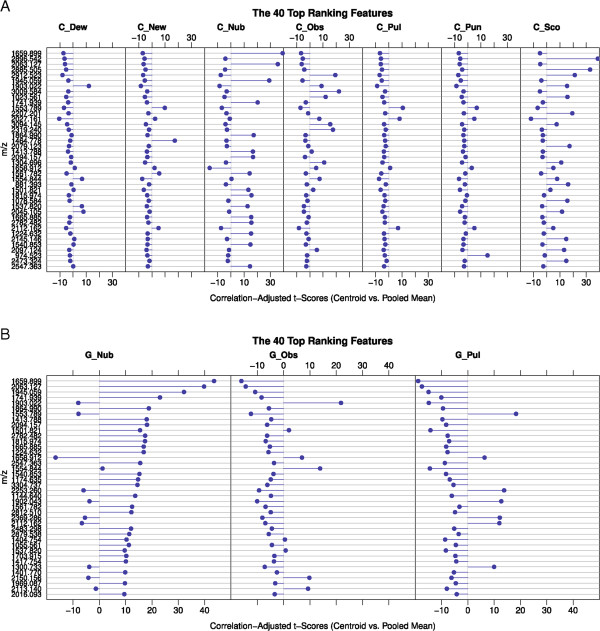
**The top 40 ranked peaks and their corresponding CAT scores of the SDA analysis for** ***Culicoides*** **species (A) and species groups (B).** With highest ranking peaks near the top of the table, the length and direction of the horizontal blue bars indicate the CAT scores of the centroid versus the pooled mean and as such describe the influence of a certain peak in differentiating between *Culicoides* species or species groups. For example, the top-ranking peak in A contributes strongly to the separation of *C. nubeculosus* from all other species, as highlighted by the length of the bar in the respective column (large positive CAT score) and the opposite direction of the bars in the columns from the bars of the other species (negative CAT scores).

To identify discriminating peaks for the species and species groups included in this study, a shrinkage discriminant analysis (SDA) was performed resulting in a ranked peak list outlined in Figure 4A and B (top 40 are shown). The peak with the highest correlation-adjusted t-score (CAT score) and therefore showing the strongest influence in differentiating between species groups or species appears at the top of the list. Every peak in the SDA list possesses a certain discrimination potential, nevertheless, no single peak was found that has exclusive species or species group discrimination characteristics except for certain peaks found in *C. nubeculosus*.

**Figure 5:**
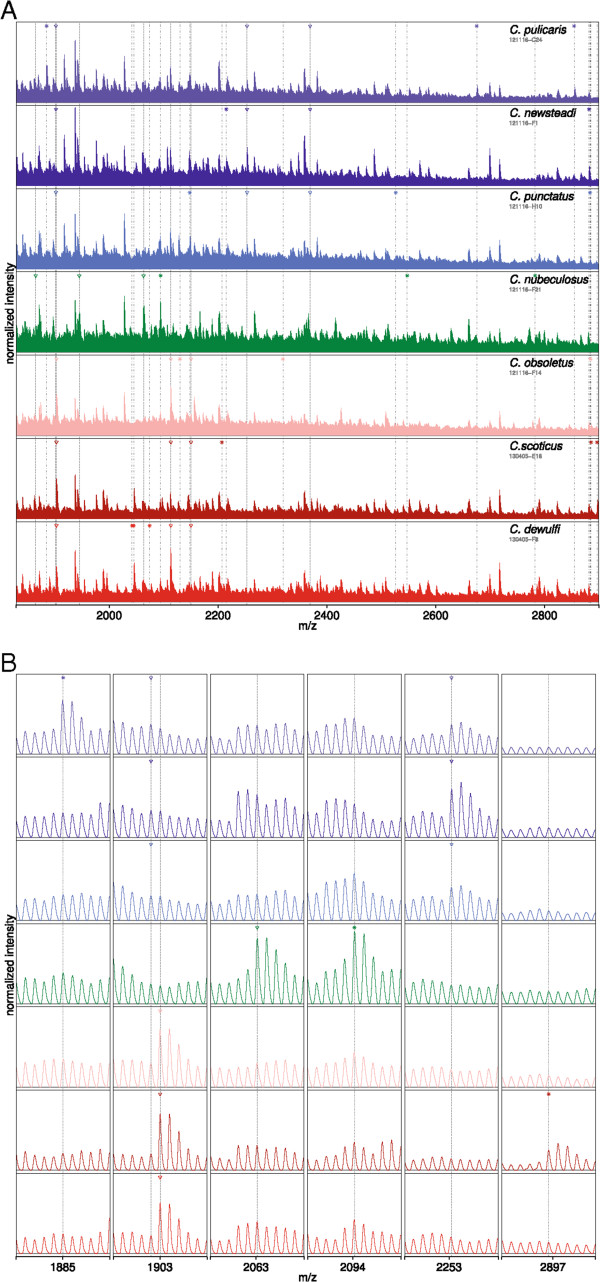
**Sections of 7 representative MALDI**-**TOF MS spectra of the 7** ***Culicoides*** **species. A**: The vertical, dash-dotted lines marked with an asterisk indicate monoisotopic peaks that are characteristic (but not exclusive) for one species (top 3 for each species). Likewise, the vertical, dashed lines marked with a triangle denote monoisotopic peaks that are characteristic for one certain species group (top 3 for each group). **B**: Zooms for six exemplary peaks. Except for the peak at 2,253.260 Da the peaks shown are all ranked under the top 40 shown in Figure 4.

A section of 7 exemplary spectra from the 7 different *Culicoides* species with marked peaks representing some of the top 3 ranked SDA features for each species or species groups was then collated (Figure 5). Not all marked peaks belong to the top 40 (Figure 4). To gain a greater insight into the resolution of the spectra and the appearance of the ranked peaks, enlarged parts of the spectra are shown in the lower portion (Figure 5).

**Figure 6:**
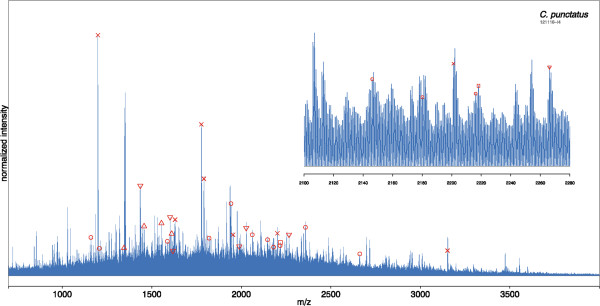
**MALDI-TOF MS spectrum of a representative specimen of** ***C. punctatus*** **.** Peaks representing the peptides of five proteins identified by offline nano-HPLC-MALDI MS/MS are indicated. For better illustration, the *m*/*z* range from 2,100 to 2,280 Da has been magnified. x = actin; â–³ = ATP-Synthase alpha subunit (mitochondrial); â–½ = ATP-Synthase beta subunit (mitochondrial); ○ = myosin; □ = tropomyosin.

Offline LC-MALDI-TOF-MS/MS and online LC-ESI-MS/MS analyses were conducted to identify peaks. 238 and 250 peptides (peptide FDR < 1%) were identified by LC-MALDI-MS and LC-ESI-MS, belonging to 21 and 22 proteins (protein FDR < 1%), respectively (data not shown). Peaks of peptides identified via these LC-MS-based analyses were matched to the corresponding peaks in the SMM spectra. In Figure 6, an example of a SMM spectrum of *C. punctatus* is shown with 28 marked peaks, all of which could be assigned to one of 5 proteins using identifications from offline LC-MALDI-TOF-MS/MS and online LC-ESI-MS/MS. Identification of the peptides by a search against the NCBI Diptera database was possible only because their respective sequences are identical to other dipteran families (e.g. *Drosophila*, *Aedes* or *Anopheles*), for which the respective sequence databases are available. An overview of the identified peptides is given in Table 2.

**Table 2:** **Identified peptides of** ***C. punctatus*** **using offline nano**-**HPLC**-**MALDI**-**TOF**-**MS**/**MS and nano**-**HPLC**-**Orbitrap**-**MS**/**MS**

		LC-MALDI				LC-Orbitrap				
Name	Sequence	MH ^+^ [Da]	Δ m[ppm]	S/N	RT[min]	MH ^+^[Da]	Δ m[ppm]	RT[min]	Modification	Identical to peptide in species of the genus:
Actin	AVFPSIVGRPR	1198.71	3.398	6835.5	39.65	1198.707	0.971	37.26		Droso., Culex, Bact., Aedes, Anoph., Belgica, Cerat.
Actin	GYSFTTTAEREIVR	1629.82	−3.862	472.8	40.02	1629.826	1.764	38.76		Maye., Culex, Culi., Bact., Psoro., Chiro., Anoph., Ochler., Wyeo., Simu., Teleo.
Actin	SYELPDGQVITIGNER	1790.89	2.934	1177.2	50.08	1790.897	2.800	47.17		Anas., Chiro., Teleo., Bact., Culi., Anoph., Calli., Simu., Psoro., Manso., Pseudo., Droso., Aedes, Belgica, Culex, Cerat.
Actin	VAPEEHPVLLTEAPLNPK	1954.06	28.501	1477.5	43.24	1954.068	1.648	42.42		Anoph., Lirio., Droso., Culex, Bact., Aedes, Belgica, Cerat.
Actin	DLYANSVLSGGTTMYPGIADR	2201.05	17.826	285.1	53.18	2201.064	4.359	53.34		Anas., Bact., Anoph., Droso., Aedes, Culex
Actin	TTGIVLDSGDGVSHTVPIYEGYALPHAILR	3151.64	23.668	645.9	56.12	3151.655	4.088	54.96		Simu., Clui., Anoph., Psoro., Culex, Ochler., Manso., Toxor., Chiro., Lirio., Teleo., Bact., Aedes, Droso.
ATP-Synthase alpha unit (mit.)	VSVREPMQTGIK	1344.73	−2.340	217.4	28.05	1344.731	0.669	26.87		Cerat., Anoph., Aedes., Culex, Droso.
ATP-Synthase alpha unit (mit.)	TALAIDTIINQQR	1456.81	−19.470	171.9	48.78	1456.819	5.182	48.99		Aedes aegypti
ATP-Synthase alpha unit (mit.)	EAYPGDVFYLHSR	1553.74	29.590	374.7	43.20	1553.742	2.022	42.11		Droso., Anoph., Culex., Aedes, Cerat.,
ATP-Synthase alpha unit (mit.)	TGAIVDVPVGDELLGR	1610.88	11.787	158.8	53.04	1610.880	3.067	53.34		Droso., Aedes, Cerat.
ATP-Synthase beta unit (mit.)	FTQAGSEVSALLGR	1435.75	−61.429	314.7	48.38	1435.758	2.830	48.06		Droso., Sarco., Cerat., Aedes
ATP-Synthase beta unit (mit.)	VALVYGQMNEPPGAR	1601.81	8.391	454.4	38.77	1601.816	3.312	37.69		Droso., Sarco., Cerat. Aedes
ATP-Synthase beta unit (mit.)	VALVYGQMNEPPGAR	1617.81	−13.445	137.7	32.82	1617.808	1.453	31.54	M8 (Oxidation)	Droso., Aedes, Cerat.
ATP-Synthase beta unit (mit.)	AIAELGIYPAVDPLDSTSR	1988.03	29.102	87.8	55.50	1988.042	4.384	55.69		Droso., Cerat., Culex, Aedes, Anoph.
ATP-Synthase beta unit (mit.)	VLDTGYPIRIPVGAETLGR	2027.13	−3.416	395.8	52.22	2027.133	2.086	52.20		Droso., Cerat.
ATP-Synthase beta unit (mit.)	IPSAVGYQPTLATDMGTMQER	2266.08	−54.675	220.4	48.05	2266.094	4.281	48.07		Drosophila virilis
Myosin	LSIENSDLLR	1159.63	31.037	191.0	43.16	1159.632	0.509	42.42		Droso., Anoph., Aedes, Culex
Myosin	AFDKIIGEWK	1206.65	−41.827	234.5	48.73	1206.652	−0.017	44.71		Anoph., Droso., Culex, Bact., Aedes, Cerat.
Myosin	VRELENELDGEQR	1586.78	16.930	273.4	29.40	1586.777	−0.266	27.03		Droso., Cerat.
Myosin	LKGAYEEGQEQLEAVR	1819.92	−49.529	476.0	34.48	1819.919	0.008	32.13		Droso., Culex, Cerat., Anoph., Aedes, Bact.
Myosin	NLADEVKDLLDQIGEGGR	1941.99	−10.703	46.7	64.00	1941.996	4.029	63.64		Droso., Culex, Anoph., Aedes, Cerat., Bact.
Myosin	LKVDDLAAELDASQKECR	2061.03	−0.848	353.1	45.20	2061.038	0.836	42.54	C17 (Carbamidomethyl)	Droso., Culex, Cerat., Anoph., Aedes, Bact.
Myosin	AKLEQTLDELEDSLEREK	2146.09	−0.126	166.2	57.37	2146.094	2.959	54.49		Dros., Culex, Cerat., Aedes, Anoph.
Myosin	TALLDSLSGEKGALQEYQEK	2180.11	−47.922	94.5	46.60	2180.114	2.789	46.41		Anoph., Culex, Aedes
Myosin	GSLEDQVVQTNPVLEAFGNAK	2216.12	18.550	83.9	56.62	2216.123	1.770	56.65		Droso., Anoph., Cerat.
Myosin	AQQELEEAEERADLAEQAISK	2358.14	−49.243	841.3	47.22	2358.148	2.661	47.08		Droso., Cerat.
Myosin	AQQELEEAEERADLAEQAISKFR	2661.31	25.032	37.3	53.59	2661.323	4.107	53.49		Droso., Cerat.
Tropomyosin	SLADEERMDALENQLKEAR	2218.08	26.636	46.3	44.13	2218.081	1.916	43.17		Chiro., Culex

Table contains only those identified peptides visible as a peak in the SMM spectrum obtained using unfractionated extracts. Droso. : Drosophila, Bact.: Bactocera, Anoph.: Anopheles, Cerat.: Ceratitis, Maye.: Mayetiola, Culi.: Culicoides, Psoro.: Psorophora, Chiro.: Chironomus, Ochler.: Ochlerotatus, Wyeo.: Wyeomyia, Simu.: Simulium, Teleo.: Teleopsis, Anas.: Anastrepha, Calli.: Calliphora, Manso.: Mansonia, Toxor.: Toxorhynchites, Pseudo.: Pseudodiamesa, Lirio.: Liriomyza, Sarco.: Sarcophaga.

## Discussion

In previous studies, MALDI-MS-based determination of species affiliation for specimens from different arthropod families had usually been performed on the basis of protein extracts using IPP [34–41]. For *Culicoides* species, Kaufmann *et al*. [42] have recently demonstrated the validity of this approach. Discrimination of different *Culicoides* species by MALDI-TOF-MS is also possible as shown in the current study using tryptic peptides derived from extracts of whole specimens. At first glance the two methods may seem to be redundant since they rely on the same starting material (an unfractionated extract) and evaluation of the resulting complex mass spectra; however, it is important to note that the spectral data used for analysis is derived from two different subsets of the proteome. Whereas IPP relies on (naturally occurring) low molecular weight proteins, SMM spectra are based on peptides from only the most abundant proteins (which are not present in IPP spectra as they generally have sizes exceeding the MALDI-TOF-MS detection limit).

**Figure 7:**
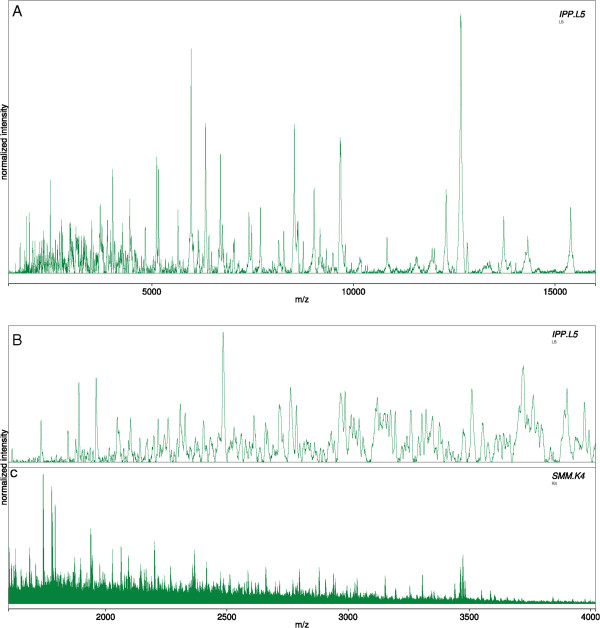
**IPP spectra vs. SMM spectra of** ***C. obsoletus*** **. A**: Complete IPP spectrum of a *C. nubeculosus* specimen from *m*/*z* 1.6-16 kDa with ca. 200 detectable peaks. **B**: A zoom of the *m*/*z* range from 1,600-4,020 Da of the IPP spectrum is depicted. This corresponds to the range where the IPP and SMM spectra overlap, ca. 100 peaks are detectable. **C**: The same range shown for an SMM spectrum of a specimen of the same species; ca. 260 peaks are detectable.

While involving additional sample preparation steps, SMM theoretically offers a much higher potential for discrimination between species due to greater resolution and higher sensitivity to single amino acid substitutions. This was practically confirmed in the current study by spectra of a *C. nubeculosus* specimen that yielded 429 peaks on average by SMM (a representative section shown in Figure 7C) when only 200 were detected on average in the corresponding IPP spectra (exemplary spectrum shown in Figure 7A). Furthermore, recording spectra of tryptic peptides derived from larger *Culicoides* proteins yielded fairly robust results for technical as well as biological replicates. In contrast, IPP spectra are known to behave in a less reproducible manner, since ionization of proteins in the higher molecular range (>10 kDa) is generally less efficient and prone to signal suppression. This may be due to variations in matrix-crystallization and co-crystallization of the sample [69] and also to the presence of small, but varying, amounts of contaminants that cannot be eliminated by the sample preparation procedure (an ion-suppression effect).

From 69 initially prepared *Culicoides* specimens, 64 were analyzed via MALDI-TOF-MS. For the five remaining specimens, species affiliation could either not be determined by PCR, the PCR and entomological analyses provided contradictory results or the midges turned out to be blood-fed (and were excluded from this study on the assumption that a significant number of detectable peptides would stem from host proteins). One reason for the difficulty in species determination via PCR, at least for some cases, seems to be the inbuilt amplification step, which renders this method susceptible to traces of contaminating material. However, in order to avoid this problem and also to extend the applicability of the SMM method to blood-fed specimens, sample preparation could be modified to generally include a dissection step for removal of the insect’s abdomen before preparing extracts, as had been reported by Kaufmann *et al*. (2012) [42].

For the final analyses, monoisotopic peak detection was carried out using a SNR cutoff value of 2, since focusing on the more intensive peaks (SNR > 3) led to less differentiability between the species (data not shown). Including peaks of lower intensity evidently seems to be important for the discrimination of *Culicoides* species, which is solely a result of the increased number of peaks available for analysis. As can be seen in Figure 6, in addition to peaks detected using a SNR > 2, each SMM spectrum is densely packed with smaller, evenly spaced peaks, however, these do not constitute electronically-induced noise. Instead, they represent statistically distributed peptides of low abundance with (isotopically) overlapping masses, reflecting the high complexity of the proteomic sample. For any given peak standing out from this peptide noise (i.e., with a SNR of 2 or higher), it can be assumed that the intensity primarily originates from one specific peptide whereas noise is minor. However, the presence of additional peptides with the same mass may compromise the ability to create unambiguous MS/MS spectra for peptides at any given m/z value.

Since it is almost impossible to record high quality MS/MS spectra from complete (and therefore highly complex) proteomic samples using only MALDI-TOF-MS/MS without prior sample fractionation, we included a chromatographic separation step in the LC-MALDI-TOF-MS/MS setup. While we were able to identify numerous peptides, these stemmed from just five different proteins that are known to be highly abundant multicellular organisms. Identification in this case was possible only because these peptides were strictly conserved with respect to distantly related dipteran species including *Drosophila* or *Anopheles*. Availability of a *Culicoides* genomic database would ensure a larger number of identifications; however, for the identification of peptide sequences varying between different *Culicoides* species, genomic information for several of the respective species would be required. In general, if no adequate databases exist, discrimination of closely related species could be enabled via limited sequencing of cDNA libraries. With messages from abundantly expressed proteins contributing most to this sequence database, identification of prominent tryptic peptides via fragmentation in an LC ESI-MS/MS analysis can be expected to yield at least some singular, species-specific entries.

To evaluate the peak matrix with a different, independent method, a PCA analysis was performed. We found that distinction between different species groups as well as individual species was possible. A reason for the incomplete separation of certain species in Figure 3B and C may stem from a closer relationship between them. For example, the smallest amount of base exchanges detectable between the COX1 consensus sequences of any two species was found for *C. obsoletus* and *C. scoticus*, which were practically inseparable in the PCA analysis. Although there is no doubt that these two constitute distinctive species, we do not have enough sequence information to make an unequivocal statement about their phylogeny based on nucleotide or amino acid differences. In summary, discrimination between different species using principal component analysis resulted in outcomes comparable to those achieved by cluster analysis. Nevertheless, no more than two different species should be included in one analytical run, since the increasing complexity of the dataset is not compatible with reduction to and graphical representation by only two principal components. In order to avoid the limitations of this approach, we decided to rely on cluster analysis for further data evaluation.

Despite several attempts in the past to establish phylogenetic status for the different *Culicoides* species, these are still a matter of debate. Different *Culicoides* species groups were analyzed and their phylogenetic relationships deduced via internal transcribed spacer (ITS1 or ITS2) [13–17] or mtDNA COX1 sequences [9–12]. Several trees based on this limited genetic data were published, showing similarities, but also differences in kinship which might have been caused by differences in the cluster algorithm, species selection, gene, or sequence length [8, 9, 14–17]. While it is reasonable to assume that some species are more closely related than others and thus form groups, there is still uncertainty about which species should be considered to belong to a species complex as well as about the relationships within already established complexes or groups. As is the case for most arthropod families, the scarcity of genomic data precludes establishing reliable phylogenetic relationships and schematic molecular trees for *Culicoides* such as those available for *Drosophila* at Flybase [70] or The database on Taxonomy of *Drosophilidae* [71]. Currently, a first step to establish a genomic database for *Culicoides* is being taken by the Genetics and Genomics group of the Pirbright Institute, where the *C. sonorensis* genome is being analyzed [72].

From the partial sequences of the mitochondrial COX1 gene (alignment shown in Additional file 4 Figure S3) that were obtained in order to identify the midges, we were also able to derive a cluster dendrogram based on sequence similarity (Figure 3). This PCR-based tree, as well as the MS-based tree substantiates the assignment of the different species into the pulicaris and the obsoletus group. According to a recently published genetic analysis of three different loci, *C. dewulfi* had been suggested not to be considered a member of the obsoletus complex [73]. Since the PCR-based dendrogram, the MS-based dendrogram as well as the PCA analysis imply a fairly close relationship between *C. dewulfi* and *C. obsoletus*/*C. scoticus*, our results do not suggest the exclusion of *C. dewulfi* from the obsoletus group. However, the low bootstrap values for the nodes close to the root of the COX1 sequence-based dendrogram do not sufficiently support the arrangement of the branches. Hence, it is difficult to assess its accuracy with respect to the implied phylogeny.

The two species *C. scoticus* and *C. obsoletus* are considered indistinguishable by morphology based on their wing pattern. Nevertheless, a recent morphometrical analysis based on 4–15 variables concluded that these two species can be discriminated from each other [7]. In contrast to the results from this study and our own PCR-based results, it was not possible to distinguish between *C. obsoletus* and *C. scoticus* using MALDI-TOF-MS data. This could be explained by the small number of individuals of *C. scoticus*. Since the two species were only distinguishable via PCR analysis and sequencing, it was not possible to select a defined number of specimens of these two species. Although our limited MS data precluded discrimination, we predict the feasibility of a proteomic approach using more specimens and thus more SMM spectra of *C. scoticus*, as had been shown by Kaufmann *et al*. using IPP [42]. A much better distinction between *C. obsoletus* and *C. scoticus* could be obtained after filtering out peaks that were present in less than 1/3 of the spectra of each of the seven species and performing a hierarchical cluster analysis based on the filtered peak tables (data not shown). One has to take into account that the filter was not applicable for *C. scoticus*, since only two respective specimens could be identified for this study. According to our present results, with a larger number of samples it should be possible to create a master peak list for each species that could be used to instantly identify unknown specimens by their SMM-spectra in a manner analogous to the workflow that had been implemented for IPP-spectra in a commercial application (SARAMIS™, AnagnosTec, Potsdam).

The genetic analysis resulted in the separation of C_pul_5 from the other specimens of *C. pulicaris* (Figure 2A). The divergent sequence is nearly identical (99%) to the sequence of a cryptic species, provisionally named *C. pulicaris P3*, which has recently been identified [8], and is sufficiently different from those of the other species studied here (Additional file 4 Figure S3). Using MALDI-MS, a discrimination of the two sister taxons could not be achieved. Apart from a possibly higher degree of relatedness, the reason for this could be the insufficient number of specimens belonging to *C. pulicaris P3*. Further investigation with a higher number of specimens is needed to show whether *C. scoticus* and *C. obsoletus* or the two sibling species of *C. pulicaris* can be differentiated from each other.

## Conclusions

In the present study, we demonstrate that MALDI-TOF-MS reliably discriminates between Palearctic *Culicoides* vector species. Furthermore, it provides a cost-effective method that allows a rapid high-throughput processing of samples. Possibly due to the low number of available specimens, the closely related species *C. scoticus* and *C. obsoletus* and the two sister taxons of *C. pulicaris* detected in this study could not be distinguished. We have shown that PCR- and SMM-analyses can be performed from the same extract of a biting midge without the necessity for previous dissection. The complete analysis is reproducible using MALDIquant, an R-based tool for analysis of mass spectrometry data. Several peptides strictly conserved between certain mosquito or fly species and *Culicoides* species could be identified via MALDI-MS/MS after previous separation by nano-HPLC. Although we were also able to obtain several MS/MS spectra for peptides with at least some species-discriminating potential, these could not be correlated to known peptide sequences, the most probable reason for this being that the available databases do not comprise *Culicoides*-specific (and thus species-specific) gene or protein sequences. With a complete *Culicoides* genomic dataset becoming available in the near future, a substitution-tolerant database search should at least ameliorate this situation.

## Electronic supplementary material

Additional file 1: Table S1: Primer sequences for COX1 – region. ^*^ see Dallas *et al*. (DOCX 16 KB)

Additional file 2: Figure S1: A scatter plot of the monoisotopic mass and corresponding decimal place of all peptides detected by MALDIquant in the *Culicoides* spectra. Peaks represented by a cross lie outside the tolerance range (±0.2 u from regression line) and were excluded from further analysis. HDPR: half decimal place rule. (ZIP 409 KB)

Additional file 3: Figure S2: Comparison of 7 MALDI-MS spectra, *m*/*z* 700–4020. Each spectrum was obtained from one representative specimen of one *Culicoides* species used in this study. (ZIP 1 MB)

Additional file 4: Figure S3: Alignment of COX1 genomic sequences. Each sequence is derived from one representative specimen of one *Culicoides* species used in this study. The unique sequence obtained from the specimen of the cryptic species *C. pulicaris P3* (specimen C_pul_5) has also been included. For better comparison, the consensus sequence is shown; nucleic acids (NAs) shown in pink in the respective sequences coincide with arbitrary positions in the reference sequence. (ZIP 96 KB)

## Abbreviations

MALDI: Matrix-assisted laser desorption ionization
ESI: Electrospray ionization
TOF: Time-of-flight
MS: Mass spectrometry
MS/MS: Tandem mass spectrometry
HPLC: High-performance liquid chromatography
IPP: Intact protein profiling
SMM: Shotgun mass mapping
PCA: Principal component analysis
HDPR: Half decimal place rule
PCR: Polymerase chain reaction
ITS: Internal transcribed spacer
COX1: Cytochrome c oxidase 1
AHSV: African horse sickness virus
BTV: Bluetongue virus
SBV: Schmallenberg virus
SNR: Signal-to-noise ratio.

## References

[1] Hoffmann B, Bauer B, Bauer C, Batza HJ, Beer M, Clausen PH, Geier M, Gethmann JM, Kiel E, Liebisch G, Liebisch A, Mehlhorn H, Schaub GA, Werner D, Conraths FJ (2009). Monitoring of putative vectors of bluetongue virus serotype 8, Germany. Emerg Infect Dis.

[2] Elbers AR, Loeffen WL, Quak S, de Boer-Luijtze E, van der Spek AN, Bouwstra R, Maas R, Spierenburg MA, de Kluijver EP, van Schaik G, van der Poel WH (2012). Seroprevalence of Schmallenberg virus antibodies among dairy cattle, the Netherlands, winter 2011–2012. Emerg Infect Dis.

[3] Wilson AJ, Mellor PS (2009). Bluetongue in Europe: past, present and future. Philos Trans R Soc Lond B Biol Sci.

[4] Carpenter S, Groschup M, Garros C, Felippe-Bauer ML, Purse B (2013). *Culicoides* biting midges, arboviruses and public health in Europe. Antiviral Res.

[5] Meiswinkel R, Baldet T, de Deken R, Takken W, Delecolle JC, Mellor PS (2008). The 2006 outbreak of bluetongue in northern Europe–the entomological perspective. Prev Vet Med.

[6] Goffredo M, Meiswinkel R (2004). Entomological surveillance of bluetongue in Italy: methods of capture, catch analysis and identification of *Culicoides* biting midges. Vet Ital.

[7] Augot D, Sauvage F, Jouet D, Simphal E, Veuille M, Couloux A, Kaltenbach ML, Depaquit J (2010). Discrimination of *Culicoides obsoletus* and *Culicoides scoticus*, potential bluetongue vectors, by morphometrical and mitochondrial cytochrome oxidase subunit I analysis. Infect Genet Evol.

[8] Pages N, Munoz-Munoz F, Talavera S, Sarto V, Lorca C, Nunez JI (2009). Identification of cryptic species of *Culicoides* (Diptera: Ceratopogonidae) in the subgenus *Culicoides* and development of species-specific PCR assays based on barcode regions. Vet Parasitol.

[9] Nolan DV, Carpenter S, Barber J, Mellor PS, Dallas JF, Mordue Luntz AJ, Piertney SB (2007). Rapid diagnostic PCR assays for members of the *Culicoides obsoletus* and *Culicoides pulicaris* species complexes, implicated vectors of bluetongue virus in Europe. Vet Microbiol.

[10] Dallas JF, Cruickshank RH, Linton YM, Nolan DV, Patakakis M, Braverman Y, Capela R, Capela M, Pena I, Meiswinkel R, Ortega MD, Baylis M, Mellor PS, Mordue (Luntz) AJ (2003). Phylogenetic status and matrilineal structure of the biting midge, *Culicoides imicola*, in Portugal, Rhodes and Israel. Med Vet Entomol.

[11] Pages N, Sarto IMV (2005). Differentiation of *Culicoides obsoletus* and *Culicoides scoticus* (Diptera: Ceratopogonidae) based on mitochondrial cytochrome oxidase subunit I. J Med Entomol.

[12] Linton YM, Mordue (Luntz) AJ, Cruickshank RH, Meiswinkel R, Mellor P, Dallas JF (2002). Phylogenetic analysis of the mitochondrial cytochrime oxidase subunit I gene of five species of the *Culicoides imicola* species complex. Med Vet Entomol.

[13] Cetre-Sossah C, Baldet T, Delecolle JC, Mathieu B, Perrin A, Grillet C, Albina E (2004). Molecular detection of *Culicoides spp*. and *Culicoides imicola*, the principal vector of bluetongue (BT) and African horse sickness (AHS) in Africa and Europe. Vet Res.

[14] Mathieu B, Perrin A, Baldet T, Delecolle JC, Albina E, Cetre-Sossah C (2007). Molecular identification of Western European species of obsoletus complex (Diptera: Ceratopogonidae) by an internal transcribed spacer-1 rDNA multiplex polymerase chain reaction assay. J Med Entomol.

[15] Perrin A, Cetre-Sossah C, Mathieu B, Baldet T, Delecolle JC, Albina E (2006). Phylogenetic analysis of *Culicoides* species from France based on nuclear ITS1-rDNA sequences. Med Vet Entomol.

[16] Gomulski LM, Meiswinkel R, Delecolle JC, Goffredo M, Gasperi G (2006). Phylogeny of the subgenus *Culicoides* and related species in Italy, inferred from internal transcribed spacer 2 ribosomal DNA sequences. Med Vet Entomol.

[17] Meiswinkel R, Gomulski LM, Delecolle JC, Goffredo M, Gasperi G (2004). The taxonomy of Culicoides vector complexes - unfinished business. Vet Ital.

[18] *Barcode of Life*. [http://www.barcodeoflife.org/]

[19] Cetre-Sossah C, Mathieu B, Setier-Rio ML, Grillet C, Baldet T, Delecolle JC, Albina E (2008). Development and evaluation of a real-time quantitative PCR assay for *Culicoides imicola*, one of the main vectors of bluetongue (BT) and African horse sickness (AHS) in Africa and Europe. Res Vet Sci.

[20] Balczun C, Vorsprach B, Meiser CK, Schaub GA (2009). Changes of the abundance of *Culicoides obsoletus s.s*. and *Culicoides scoticus* in Southwest Germany identified by a PCR-based differentiation. Parasitol Res.

[21] Schwenkenbecher JM, Mordue AJ, Switek K, Piertney SB (2009). Discrimination of *Culicoides* midge larvae using multiplex polymerase chain reaction assays based on DNA sequence variation at the mitochondrial cytochrome C oxidase I gene. J Med Entomol.

[22] Stephan A, Clausen PH, Bauer B, Steuber S (2009). PCR identification of *Culicoides dewulfi* midges (Diptera: Ceratopogonidae), potential vectors of bluetongue in Germany. Parasitol Res.

[23] Wenk CE, Kaufmann C, Schaffner F, Mathis A (2011). Molecular characterization of Swiss Ceratopogonidae (Diptera) and evaluation of real-time PCR assays for the identification of *Culicoides* biting midges. Vet Parasitol.

[24] von Bergen M, Eidner A, Schmidt F, Murugaiyan J, Wirth H, Binder H, Maier T, Roesler U (2009). Identification of harmless and pathogenic algae of the genus *Prototheca* by MALDI-MS. Proteomics Clin Appl.

[25] Sauer S, Freiwald A, Maier T, Kube M, Reinhardt R, Kostrzewa M, Geider K (2008). Classification and Identification of Bacteria by Mass Spectrometry and Computational Analysis. PLoS One.

[26] Mellmann A, Bimet F, Bizet C, Borovskaya AD, Drake RR, Eigner U, Fahr AM, He Y, Ilina EN, Kostrzewa M, Maier T, Mancinelli L, Moussaoui W, Pre´vost G, Putignani L, Seachord CL, Tang YW, Harmsen D (2009). High interlaboratory reproducibility of matrix-assisted laser desorption ionization-time of flight mass spectrometry-based species identification of nonfermenting bacteria. J Clin Microbiol.

[27] Santos C, Paterson RR, Venancio A, Lima N (2010). Filamentous fungal characterizations by matrix-assisted laser desorption/ionization time-of-flight mass spectrometry. J Appl Microbiol.

[28] Sauer S, Kliem M (2010). Mass spectrometry tools for the classification and identification of bacteria. Nat Rev Microbiol.

[29] Stevenson LG, Drake SK, Shea YR, Zelazny AM, Murray PR (2010). Evaluation of matrix-assisted laser desorption ionization-time of flight mass spectrometry for identification of clinically important yeast species. J Clin Microbiol.

[30] van Veen SQ, Claas EC, Kuijper EJ (2010). High-throughput identification of bacteria and yeast by matrix-assisted laser desorption ionization-time of flight mass spectrometry in conventional medical microbiology laboratories. J Clin Microbiol.

[31] Giebel R, Worden C, Rust SM, Kleinheinz GT, Robbins M, Sandrin TR (2010). Microbial fingerprinting using matrix-assisted laser desorption ionization time-of-flight mass spectrometry (MALDI-TOF MS) applications and challenges. Adv Appl Microbiol.

[32] Caprioli G, Cristalli G, Ragazzi E, Molin L, Ricciutelli M, Sagratini G, Seraglia R, Zuo Y, Vittori S (2010). A preliminary matrix-assisted laser desorption/ionization time-of-flight approach for the characterization of Italian lentil varieties. Rapid Commun Mass Spectrom.

[33] Mazzeo MF, Giulio BD, Guerriero G, Ciarcia G, Malorni A, Russo GL, Siciliano RA (2008). Fish authentication by MALDI-TOF mass spectrometry. J Agric Food Chem.

[34] Perera MR, Vargas RDF, Jones MGK (2005). Identification of aphid species using protein profiling and matrix-assisted laser desorption/ionization time-of-flight mass spectrometry. Entomol Exp Appl.

[35] Campbell PM (2005). Species differentiation of insects and other multicellular organisms using matrix-assisted laser desorption/ionization time of flight mass spectrometry protein profiling. Syst Entomol.

[36] Feltens R, Gorner R, Kalkhof S, Groger-Arndt H, von Bergen M (2010). Discrimination of different species from the genus Drosophila by intact protein profiling using matrix-assisted laser desorption ionization mass spectrometry. BMC Evol Biol.

[37] Muller P, Pfluger V, Wittwer M, Ziegler D, Chandre F, Simard F, Lengeler C (2013). Identification of cryptic Anopheles mosquito species by molecular protein profiling. PLoS One.

[38] Karger A, Kampen H, Bettin B, Dautel H, Ziller M, Hoffmann B, Suss J, Klaus C (2012). Species determination and characterization of developmental stages of ticks by whole-animal matrix-assisted laser desorption/ionization mass spectrometry. Ticks Tick Borne Dis.

[39] Yssouf A, Flaudrops C, Drali R, Kernif T, Socolovschi C, Berenger JM, Raoult D, Parola P (2013). Matrix-assisted laser desorption ionization-time of flight mass spectrometry for rapid identification of tick vectors. J Clin Microbiol.

[40] Yssouf A, Socolovschi C, Flaudrops C, Ndiath MO, Sougoufara S, Dehecq JS, Lacour G, Berenger JM, Sokhna CS, Raoult D, Parola P (2013). Matrix-assisted laser desorption ionization–time of flight mass spectrometry: an emerging tool for the rapid identification of mosquito vectors. PLoS One.

[41] Hoppenheit A, Murugaiyan J, Bauer B, Steuber S, Clausen PH, Roesler U (2013). Identification of Tsetse (Glossina spp.) using matrix-assisted laser desorption/ionisation time of flight mass spectrometry. PLoS Negl Trop Dis.

[42] Kaufmann C, Schaffner F, Ziegler D, Pfluger V, Mathis A (2012). Identification of field-caught *Culicoides* biting midges using matrix-assisted laser desorption/ionization time of flight mass spectrometry. Parasitology.

[43] Dekker LJ, Boogerd W, Stockhammer G, Dalebout JC, Siccama I, Zheng P, Bonfrer JM, Verschuuren JJ, Jenster G, Verbeek MM, Luider TM, Sillevis Smitt PA (2005). MALDI-TOF mass spectrometry analysis of cerebrospinal fluid tryptic peptide profiles to diagnose leptomeningeal metastases in patients with breast cancer. Mol Cell Proteomics.

[44] Villanueva J, Philip J, Chaparro CA, Li Y, Toledo-Crow R, DeNoyer L, Fleisher M, Robbins RJ, Tempst P (2005). Correcting Common Errors in Identifying Cancer-Specific Serum Peptide Signatures. J Proteome Res.

[45] Schmidt F, Fiege T, Hustoft HK, Kneist S, Thiede B (2009). Shotgun mass mapping of *Lactobacillus* species and subspecies from caries related isolates by MALDI-MS. Proteomics.

[46] Jehmlich N, Schmidt F, Taubert M, Seifert J, von Bergen M, Richnow HH, Vogt C (2009). Comparison of methods for simultaneous identification of bacterial species and determination of metabolic activity by protein-based stable isotope probing (Protein-SIP) experiments. Rapid Commun Mass Spectrom.

[47] Muller SA, Kohajda T, Findeiss S, Stadler PF, Washietl S, Kellis M, von Bergen M, Kalkhof S (2010). Optimization of parameters for coverage of low molecular weight proteins. Anal Bioanal Chem.

[48] Kalkhof S, Haehn S, Ihling C, Paulsson M, Smyth N, Sinz A (2008). Determination of disulfide bond patterns in laminin beta1 chain N-terminal domains by nano-high-performance liquid chromatography/matrix-assisted laser desorption/ionization time-of-flight/time-of-flight mass spectrometry. Rapid Commun Mass Spectrom.

[49] R Core Team (2013). R: A language and environment for statistical computing.

[50] Gibb S (2013). MALDIquantForeign: Import/Export routines for MALDIquant. R package version 0.5.1.

[51] Gibb S, Strimmer K (2012). MALDIquant: a versatile R package for the analysis of mass spectrometry data. Bioinformatics.

[52] Senko MW, Beu SC, McLafferty FW (1995). Determination of monoisotopic masses and ion populations for large biomolecules from resolved isotopic distributions. J Am Soc Mass Spectrom.

[53] Park K, Yoon JY, Lee S, Paek E, Park H, Jung HJ, Lee SW (2008). Isotopic peak intensity ratio based algorithm for determination of isotopic clusters and monoisotopic masses of polypeptides from high-resolution mass spectrometric data. Anal Chem.

[54] Karty JA, Ireland MM, Brun YV, Reilly JP (2002). Artifacts and unassigned masses encountered in peptide mass mapping. J Chromatogr B Analyt Technol Biomed Life Sci.

[55] Schmidt F, Schmid M, Jungblut PR, Mattow J, Facius A, Pleissner K-P (2003). Iterative data analysis is the key for exhaustive analysis of peptide mass fingerprints from proteins separated by two-dimensional electrophoresis. J Am Soc Mass Spectrom.

[56] Gibb S (2013). Cleaver: Cleavage of polypeptide sequences. R package version 1.0.0.

[57] The UniProt Consortium (2012). Reorganizing the protein space at the Universal Protein Resource (UniProt). Nucleic Acids Res.

[58] Claesen J, Dittwald P, Burzykowski T, Valkenborg D (2012). An efficient method to calculate the aggregated isotopic distribution and exact center-masses. J Am Soc Mass Spectrom.

[59] Venables WN, Ripley BD (2002). Modern Applied Statistics with S.

[60] Dice LR (1945). Measures of the amount of ecologic association between species. Ecology.

[61] Meyer D, Buchta C (2013). Proxy: Distance and Similarity Measures. R package version 04-10.

[62] Ward JH (1963). Hierarchical grouping to optimize an objective function. J Am Stat Assoc.

[63] Oksanen J, Blanchet FG, Kindt R, Legendre P, Minchin PR, O’Hara RB, Simpson GL, Solymos P, Stevens MHH, Wagner H (2013). Vegan: Community Ecology Package. R package version 2.0-9.

[64] Ahdesmäki M, Strimmer K (2010). Feature selection in omics prediction problems using cat scores and false nondiscovery rate control. Annals Appl Statistics.

[65] Zuber V, Strimmer K (2009). Gene ranking and biomarker discovery under correlation. Bioinformatics.

[66] Ahdesmäki M, Zuber V, Gibb S, Strimmer K (2013). sda: Shrinkage Discriminant Analysis and CAT Score Variable Selection. R package version 1.3.2.

[67] Paradis E, Claude J, Strimmer K (2004). APE: Analyses of Phylogenetics and Evolution in R language. Bioinformatics.

[68] Kimura M (1980). A simple method for estimating evolutionary rates of base substitutions through comparative studies of nucleotide sequences. J Mol Evol.

[69] Albrethsen J (2007). Reproducibility in protein profiling by MALDI-TOF mass spectrometry. Clin Chem.

[70] Tweedie S, Ashburner M, Falls K, Leyland P, McQuilton P, Marygold S, Millburn G, Osumi-Sutherland D, Schroeder A, Seal R, Zhang H (2009). FlyBase: enhancing *Drosophila* Gene Ontology annotations. Nucleic Acids Res.

[71] *ToxoDros*. [http://www.taxodros.uzh.ch/]

[72] *Genetics and Genomics*. [http://www.research.pirbright.ac.uk/geneticsgenomics/]

[73] Schwenkenbecher JM, Mordue AJ, Piertney SB (2009). Phylogenetic analysis indicates that *Culicoides dewulfi* should not be considered part of the *Culicoides obsoletus* complex. Bull Entomol Res.

