# Supplementary material for: Species determination of Culicoides biting midges via peptide profiling using matrix-assisted laser desorption ionization mass spectrometry
Source: Parasit Vectors. 2014 Aug 24;7:392. doi: 10.1186/1756-3305-7-392 (PMC4158057; doi:10.1186/1756-3305-7-392)
Supplement: Supplementary file 1 — Additional file 1: Table S1: Primer sequences for COX1 – region. * see Dallas et al. (DOCX 16 KB) [file 13071_2014_1570_MOESM1_ESM.docx]

Primers^*^ for COXI-region:

C1-J-1718: 5’-GGAGGATTTGGAAATTGATTAGT-3’

C1-N-2191: 5’-CAGGTAAAATTAAAATATAAACTTCTGG-3’

(length of amplificate app. 523 bp)

**New primers for COX1-region derived from Dallas et al.:**

| PanCuli-COX1-195F | TTT ATA GTT ATR CCT ATY ATA ATT GG |  |
| --- | --- | --- |
| PanCuli-COX1-207F | ACC TAT YAT AAT TGG DGG RTT YGG |  |
| PanCuli-COX1-211F | ATC ATA ATT GGT GGG TTT GGW AAY TGA |  |
| PanCuli-COX1-220F | GGA GGA TTT GGA AAT TGA TTA GT | (=C1-J-1718) |
|  |  |  |
| PanCuli-COX1-727R | TAT AAA CTT CDG GRT GNC CAA ARA ATC |  |
| PanCuli-COX1-744R | TCC TGG TAA AAT TAA AAT RTA WAC TTC DGG | (=C1-N-2191 (mod.)) |
| PanCuli-COX1-742R | C AGG TAA AAT TAA AAT ATA AAC TTC TGG | (=C1-N-2191) |
|  |  |  |
